# Safety and efficacy of long-acting cabotegravir/rilpivirine versus standard oral antiretroviral therapy: a systematic review and meta-analysis

**DOI:** 10.1093/jac/dkae480

**Published:** 2025-01-08

**Authors:** Samuel Bungaran Partahi Saud Manalu, Andrea Perez Navarro, Cassandra Fairhead, Andrew Hill

**Affiliations:** School of Public Health, Imperial College London, London, UK; School of Medicine, Imperial College London, London, UK; Charite Universitatsmedizin Berlin, Institute of Tropical Medicine and International Health, Sudring 2-3, Berlin, Germany; Department of Pharmacology and Therapeutics, University of Liverpoool, Liverpool, UK

## Abstract

**Background:**

In 2023, there were 39.9 million people living with HIV (PLWH) worldwide and 630 000 deaths related to HIV. New strategies are needed, and long-acting antiretrovirals (LAAs) are now widely considered to have great potential to help end the HIV epidemic. This systematic review and meta-analysis compare the safety and efficacy of LAA versus standard oral treatment (SOT) for HIV.

**Methods:**

PubMed and Embase databases, supplemented by ClinicalTrials.gov and grey literature, were searched. Randomized controlled trials (RCTs) reporting efficacy and/or safety of LAA versus SOT for PLWH until June 2024 were included. Efficacy (HIV RNA < 50 copies/mL) and HIV RNA ≥ 50 copies/mL, adverse events (AEs), treatment discontinuation, CD4 count, metabolic parameters and drug resistance were assessed. Prespecified subgroup analyses were conducted. The risk of bias was assessed with Cochrane RoB 2.0. Certainty of evidence was assessed using GRADE.

**Results:**

Six RCTs were eligible for inclusion, involving 2829 participants. LAA was non-inferior to SOT in suppressing HIV RNA < 50 copies/mL [Risk Difference (RD), −0.00; 95% CI, −0.03–0.02; *P* = 0.83; I^2^ = 51%; high quality of evidence (QoE)]. LAA was associated with higher drug resistance (percentage pooled estimate, 57%; 95% CI, 33%–78% versus 9%; 95% CI, 2%–30%; moderate QoE) and risk of grade 1–4 AEs than SOT [Risk Ratio (RR), 1.22; 95% CI, 1.12–1.33; *P* < 0.001; I^2^ = 62%; moderate QoE].

**Conclusions:**

LAA has non-inferior efficacy compared to SOT. However, participants receiving LAA were at a higher risk of developing drug resistance, cross-resistance and AEs.

## Introduction

### Background

Four decades after its identification in 1984, HIV remains a major public health issue. Approximately 39.9 million people are living with HIV (PLWH) worldwide, and there were an estimated 630 000 HIV-related deaths in 2022.^1,2^ Although there has been a 38% decline in new infections since 2010 to 1.3 million new HIV acquisitions in 2022, for PLWH, the treatment coverage is still sub-optimal, with just 77% of adults and 57% of children living with HIV receiving ART. This leaves 9.2 million PLWH without access to ART.^1,2^ In efforts to end the HIV epidemic as a public health threat, addressing the challenge of unequal access to treatment must be prioritized.^3^

Lifelong medications rely heavily on user adherence. In the case of ART, nonadherence remains a significant challenge.^4^ A study conducted from 2017 to 2018 in the USA evaluated the adherence of more than 200 000 PLWH. The study revealed that over 60% had an adherence level of less than 70%, and more than 40% had an adherence level of less than 80%.^5^ Similarly, a study conducted in India found that adherence was only 81.3%, while in a tertiary hospital in Brazil, the prevalence of nonadherence was 28.4%.^6,7^ Several factors may contribute to ART nonadherence among PLWH, including stigma, discrimination, privacy concerns, side effects, lack of support and treatment fatigue.^4,8–11^

Despite significant progress in simplifying ART to a single, fixed-dose oral treatment, ongoing nonadherence suggests a need for further innovation while maintaining efficacy and tolerability.^12–14^ A promising approach to meet PLWH’s needs is the development of long-acting antiretroviral (LAA) drugs, which reduce dosing intervals from hours to weeks or even months.^12^ A key aim in the development of LAA is hoped to fundamentally improve the convenience and acceptability of ART for PLWH.^15–17^

However, despite the potential benefits, implementation of LAA is currently expensive. The price of a 2-monthly injection of cabotegravir and rilpivirine in the UK could cost approximately £9840 or $13 000 per person per year, and availability of this combination is minimal in LMICs outside of clinical trials. In comparison, the combination of tenofovir disoproxil, lamivudine and dolutegravir costs only $75 per person per year in a low- or middle-income country.^18,19^ So, comprehensively and critically evaluating LAA compared to once-daily standard oral treatment (SOT) is vital to enable policy decisions that maximize effective, safe and equitable ART delivery. It is important to ensure that these novel drugs offer true benefits and are not merely an evergreening strategy anticipating the soon-to-end patents on current SOT drugs. This systematic review and meta-analysis aimed to evaluate the efficacy and safety of LAA compared to SOT.^20,21^

## Methods

This study protocol followed the Preferred Reporting Items for Systematic Reviews and Meta-Analyses for Protocols (PRISMA-P) guidelines and was registered with PROSPERO (CRD42024553249).^22,23^

### Search strategy

The PubMed and Embase databases were systematically searched using a focused search strategy, supplemented by advanced searches in ClinicalTrials.gov and an additional grey literature source from the conference report. HIV, LAAs, efficacy, safety and resistance were the key concepts (the full search strategy is available in Appendix S1 (available as Supplementary data at *JAC* Online)). Records up to June 2024 were included.

### Inclusion criteria and PICO framework

Randomized controlled trials (RCTs) reported in English were included. The inclusion and exclusion criteria followed a PICO framework.^24^ The population was PLWH of any age, background or geographic region. Interventions analysed were LAA, including cabotegravir, lenacapavir and islatravir. The comparator was SOT. The outcomes assessed were efficacy and safety over at least 48 weeks, as assessed through viral suppression HIV RNA < 50 copies/mL and HIV RNA ≥ 50 copies/mL and the following safety outcomes: grade 1–4 adverse events (AEs); grade 1–4 AEs excluding injection site reactions (ISRs); AEs leading to treatment discontinuation; ISR rate; change in LDL; change in CD4 count; incident hypertension; change in glucose level; change in bodyweight and treatment-emergent integrase strand transfer inhibitor (INSTI) resistance. This analysis focused on INSTI and not NNRTI resistance because INSTI-based ART is the global first-line ART regimen and therefore potential INSTI cross-resistance risk requires particularly close monitoring. Meanwhile, at a global level, NNRTIs are becoming less commonly used, reflecting a reduced role in the current treatment strategy. Subgroup analyses of naïve and switch studies and between studies with or without TDF in the SOT arm were conducted.

### Data extraction and quality assessment

Data, including baseline participant demographics, were extracted into a pre-written proforma (Appendix S2). The principal author independently evaluated the risk of bias in randomized studies using the updated Cochrane tool for assessing the risk of bias in randomized trials (RoB 2.0).^25^

### Statistical analysis

Meta-analysis was performed in RevMan 5 (version 5.4.1) or, for certain outcomes, R (version 4.3.1). Efficacy and safety data for LAA were compared against SOT across all prespecified outcomes. Due to the clinical diversity in participant populations, random-effects models were used for heterogeneity (I^2^) > 50% and fixed-effect models were used for heterogeneity (I^2^) ≤ 50%. For dichotomous outcomes, the effect estimate was calculated as risk difference or risk ratio; for continuous outcomes, it was calculated as mean difference.

Heterogeneity between studies was represented using the I^2^ statistical parameter. Heterogeneity was considered unimportant if I^2^ < 40%, moderate if I^2^ lay between 40% and 60%, substantial if I^2^ lay between 50% and 90% and considerable if I^2^ > 75%.^24^ Subgroup analysis was performed to explore effect variations across several prespecified populations. Leave-one-out sensitivity analysis was performed for specific outcomes to assess whether one study significantly affected the overall effect estimate.^24^ Publication bias in key outcomes was assessed with a funnel plot and Egger’s test.^26^

### Certainty of the evidence

The certainty of evidence was assessed using the Grading of Recommendations Assessment, Development, and Evaluation (GRADE) approach.^24,27^

## Results

**Figure 1.**
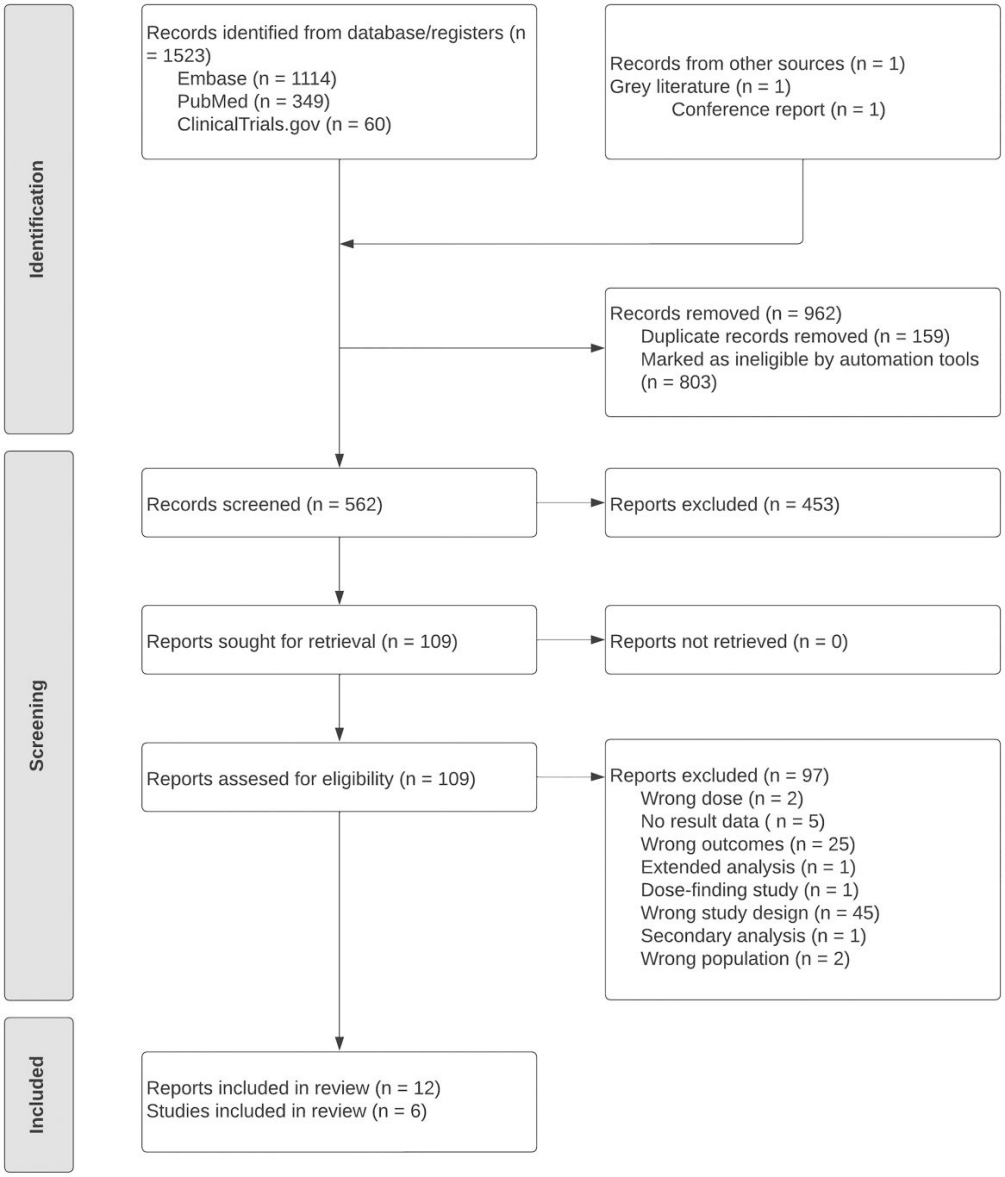
PRISMA flow diagram of the study selection process.

The systematic literature search identified 1523 records (Figure 1), including 1114 from Embase, 349 from PubMed and 60 from ClinicalTrials.gov. Additionally, one record was identified from grey literature. After removing 962 records due to duplication and ineligibility, 562 were screened based on titles and abstracts. Full texts reviewed for 109 reports. Following full-text screening, 95 reports were excluded (reasons detailed in Figure 1). In total, six RCTs from 12 reports were eligible for inclusion in the meta-analysis.^28–33^

These six RCTs were published between 2017 and 2024 and included 2829 participants aged 16–82. Overall, 30% were female, and 41% were of Black or African American ethnicity (Table S1). The length of the study ranged from 48 to 52 weeks. All RCTs other than LATTE-2 were Phase 3.^29^ Two RCTs recruited ART-naive people living with HIV (PLWH) who achieved viral suppression after oral induction therapy; three recruited PLWH who switched ART and one recruited PLWH for whom their ART had failed.^28–33^ All studies were identified as switch studies (Table 1). All six RCTs were assessed to have low bias risk based on RoB 2.0 (Table S2).

**Table 1.** Summary table of included studies

Study	Study type	Phase	Interventions	Study length^a^	LAA	SOT	Countries involved
FLAIR^28^	HIV positive—Switch (OL)	3	CAB/RPV versus DTG/ABC/3TC	48 weeks	283	283	USA, Canada, France, Germany, Italy, Japan, Netherlands, Russian Federation, South Africa, Spain and UK
LATTE-2^29^	HIV positive—Switch (OL)	2b	CAB/RPV versus CAB/ABC/3TC	48 weeks	115	56	USA, Canada, Spain, France and Germany
SOLAR^30^	HIV positive—Switch (OL)	3b	CAB/RPV versus BIC/FTC/TAF	12 months	447	223	Australia, Austria, Belgium, Canada, France, Germany, Ireland, Italy, Japan, Netherlands, Spain, Switzerland and UK
ATLAS^31^	HIV positive—Switch (OL)	3	CAB/RPV versus Oral PI or NNRTI or INSTI-based therapy	48 weeks	308	308	USA, Canada, Mexico, Argentina, France, Germany, Italy, Spain, Sweden, Russian Federation, South Africa, Republic of Korea and Australia
CARES^32^	HIV positive—Switch (OL)	3b	CAB/RPV versus TDF/3TC or FTC/DTG or FTC/NVP or FTC/EPV	48 weeks	255	257	Uganda, Kenya and South Africa
LATITUDE^33^	HIV positive—Switch (OL)	3	CAB/RPV versus all oral standard care	52 weeks	146	148	USA and Puerto Rico

OL, open label; DB, double blind; CAB, cabotegravir; RPV, rilpivirine; DTG, dolutegravir; ABC, abacavir; 3TC, lamivudine; BIC, bictegravir; FTC, emtricitabine; TAF, tenofovir alafenamide; TDF, tenofovir disoproxil fumarate; NVP, nevirapine; EPV, efavirenz; PI, protease inhibitor; NNRTI, non-nucleoside reverse transcriptase inhibitor; INSTI, integrase strand transfer inhibitor.

^a^Some trials may have continued beyond this period, but only data available around stated study length was considered.

### Efficacy of LAA compared to SOT

**Figure 2.**
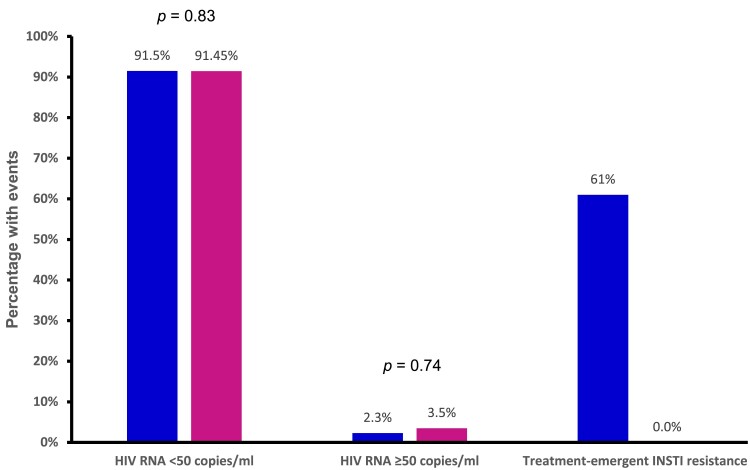
Summary of the efficacy of LAA (first bars) versus SOT (second bars). INSTI, integrase strand transfer inhibitor. Treatment-emergent INSTI resistance figures did not use total participants as the denominators but were limited to successfully genotyped participants.

**Figure 3.**
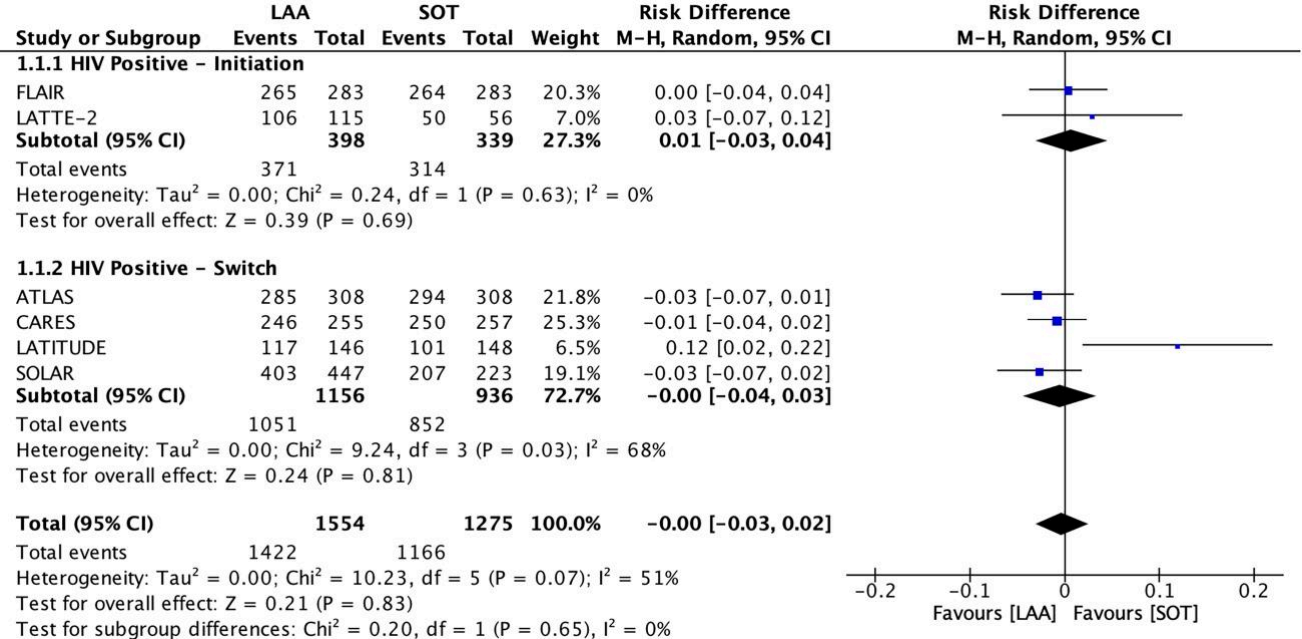
Forest plot for HIV RNA < 50** **copies/mL at Week 48, LAA versus SOT. M-H, Mantel–Haenszel; CI, confidence interval.

LAA was non-inferior to SOT in viral suppression of HIV RNA < 50 copies/mL (91.5% [1422/1554] versus 91.4% [1166/1275]; RD, −0.00; 95% CI, −0.03 to 0.02; *P* = 0.83, I^2^ = 51%; high quality of evidence (QoE)) (Figures 2 and 3 and Table S3). The lower CI (−0.03) was above the −10% non-inferiority margin.^34^ LAA was also non-inferior to SOT in HIV RNA ≥ 50 copies/mL (2.3% [36/1554] versus 3.5% [45/1275]; RD, −0.00; 95% CI, −0.03 to 0.02; *P* = 0.74, I^2^ = 85%; moderate QoE) (Figure 2, Table S3 and Figure S1). The upper CI (0.02) was below the 4% non-inferiority margin.^34^

**Figure 4.**
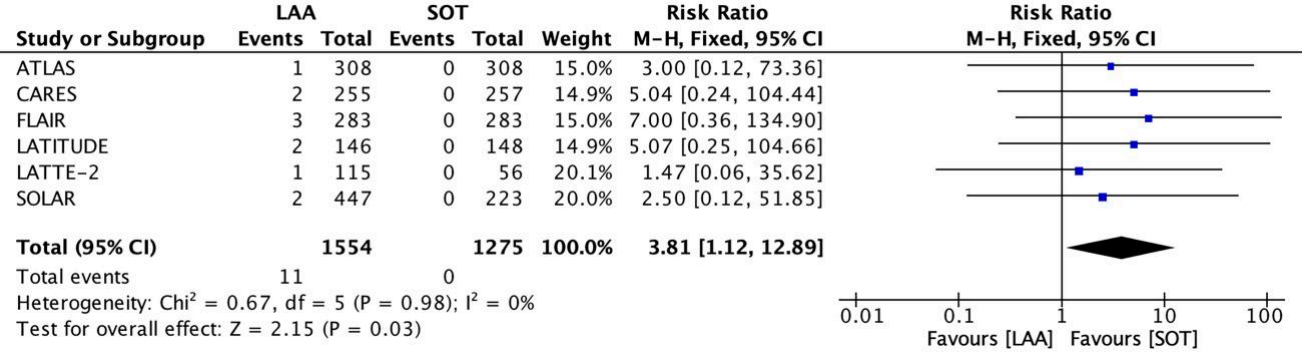
Forest plot for treatment-emergent INSTI resistance per total participants, LAA versus SOT. M-H, Mantel–Haenszel; CI, confidence interval.

LAA was associated with increased risk of treatment-emergent INSTI resistance among successfully genotyped participants with virological failure (61% [11/18] versus 0% [0/29]; [percentage pooled estimate 57%; 95% CI, 33% to 78%] versus [percentage pooled estimate, 9%; 95% CI, 2% to 30%; moderate QoE]) (Figure 2, Figure S2 and Table S3). When compared with the total number of participants in each study, LAA was significantly associated with an increased risk of treatment-emergent INSTI resistance (0.07% [11/1554] versus 0% [0/1275]; RR, 3.81; 95% CI, 1.12 to 12.89; *P* = 0.03, I^2^ = 0%) (Figure 4).

In LAA, 17 major INSTI-associated drug resistance mutations (DRMs) were detected in 13 individuals, and 5/13 (38.5%) also had intermediate to high dolutegravir cross-resistance. The most common INSTI-associated DRMs identified were Q148R (5/17, 29.4%), N155H (2/17, 11.8%) and Q148K (2/17, 11.8%) (Table S4).

### Safety of LAA compared to SOT

**Figure 5.**
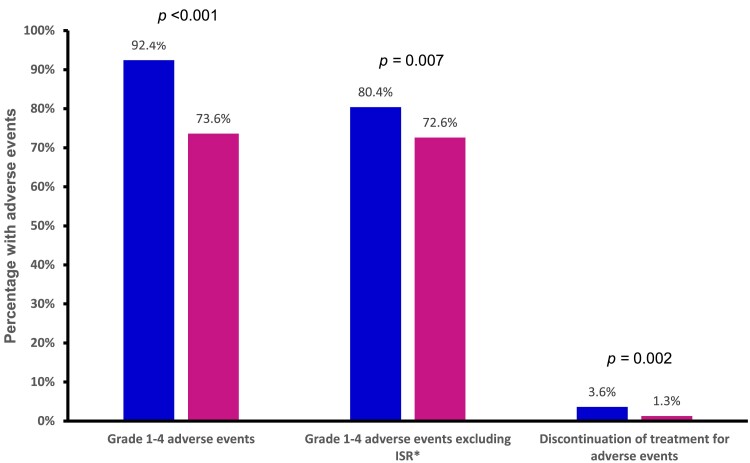
Summary of the safety of LAA (first bars) versus SOT (second bars) as a treatment. *Data from LATTE-2 were not provided.

LAA was associated with significantly increased risk of grade 1–4 AEs (92.4% versus 73.6%; RR, 1.22; 95% CI, 1.12 to 1.33; *P* < 0.001; I^2^ = 83%; moderate QoE), grade 1–4 AEs excluding ISR (80.4% versus 72.6%; RR, 1.10; 95% CI, 1.03 to 1.19; *P* = 0.007; I^2^ = 62%; moderate QoE) and AEs leading to treatment discontinuation (3.6% versus 1.3%; RR, 2.58; 95% CI, 1.43 to 4.67; *P *= 0.002; I^2^ = 34%; high QoE) (Figure 5, Table S3 and Figures S3–S5). The percentage of ISR among participants receiving LAA was 79% (1108/1408) (meta-analysis pooled estimate, 83%; 95% CI, 71% to 90%; I^2^ = 91%; low QoE) (Figure S6 and Table S3).

Compared to SOT, LAA was associated with a significantly increased LDL level by 0.22 mmol/L (MD, 0.22; 95% CI, 0.06 to 0.38; *P *= 0.008; I^2^ = 75%; moderate QoE), lower CD4+ cell rise by 21.6 cells/mm^3^ (MD, −21.6; 95% CI, −37.97 to −5.24; *P* = 0.01; I^2^ = 36%; high QoE) and increased risk of hypertension by 75% (4.3% versus 2.5%; RR, 1.75; 95% CI, 1.07 to 2.87; *P* = 0.03; I^2^ = 0%; high QoE) (Table S3 and Figures S7–S9).

No significant difference was observed for glucose level (MD, 0.03; 95% CI, −0.06 to 0.13; *P* = 0.48; I^2^ = 0%; high QoE) and body weight change (MD, 0.94; 95% CI, −0.14 to 2.02; *P *= 0.09; I^2^ = 89%; moderate QoE) (Table S3 and Figures S10 and S11).

### Subgroup and sensitivity analysis

**Figure 6.**
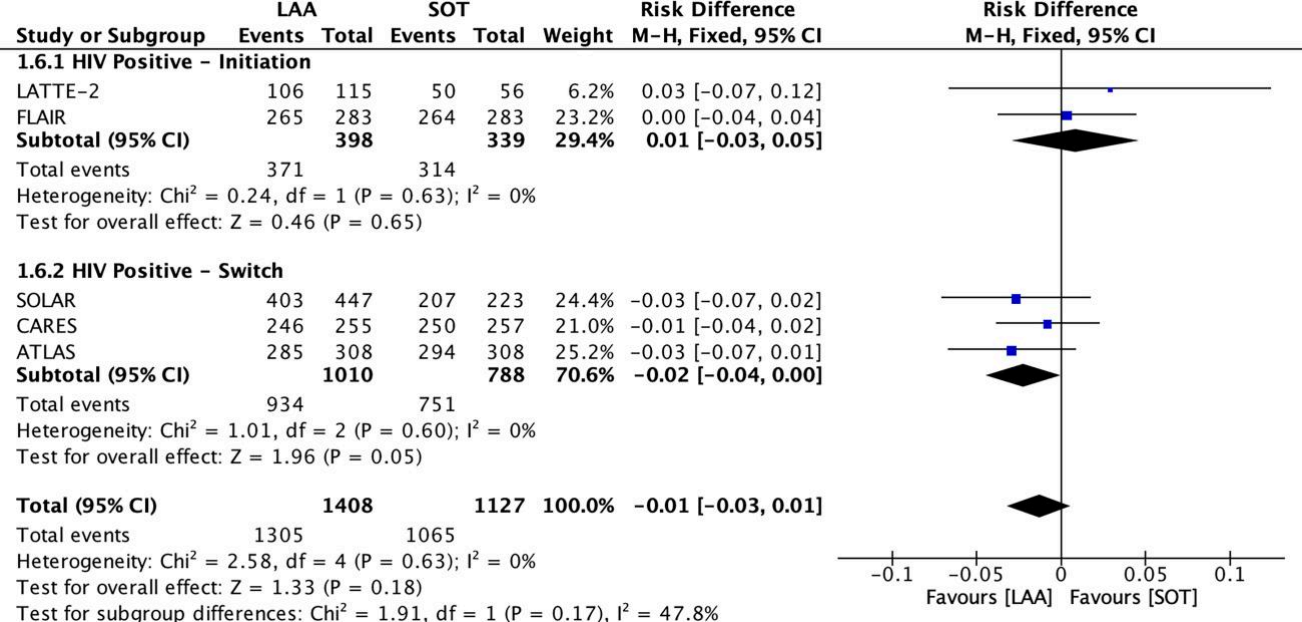
Forest plot for sensitivity analysis of HIV RNA < 50 copies/mL at Week 48 by taking out the LATITUDE study, LAA versus SOT. M-H, Mantel–Haenszel; CI, confidence interval.

**Figure 7.**
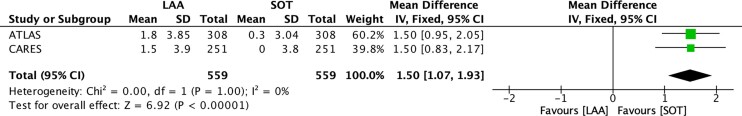
Forest plot for sensitivity analysis of change in weight by taking out FLAIR study, LAA versus SOT. M-H, Mantel–Haenszel; CI, confidence interval.

No significant differences were identified between LAA and SOT for initiation and switch studies for HIV RNA < 50 copies/mL (*P = *0.65) and HIV RNA ≥ 50 copies/mL (*P* = 0.29) (Figure 3 and Figure S1). There was also no difference between studies with or without TDF in the SOT arm on the risk of hypertension (*P *= 0.73) (Figure S9). Sensitivity analysis showed that LATITUDE was an influential study. While the risk difference was similar, the heterogeneity dropped (92.7% [1305/1408] versus 94.5% [1065/1127]; RD, −0.01; 95% CI, −0.03 to 0.01; *P* = 0.18, I^2^ = 0%) (Figure 6). However, we did not exclude LATITUDE in order to best reflect real-world data and to prevent publication bias. In a sensitivity analysis of body weight change, we excluded FLAIR as this was the only study analysed without TDF. This sensitivity analysis showed that participants in studies with TDF had a significantly greater risk of weight gain by 1.5 kg (MD, 1.50; 95% CI, 1.07 to 1.93; *P *< 0.001; I^2^ = 0%) (Figure 7).

### Risk of bias, GRADE assessment and publication bias

Five RCTs had a low risk of bias, and one was categorized as having some concerns. This was due to an open-label RCT with a lack of information about whether a deviation from the intended intervention arose because of the trial context (Table S2). Using GRADE criteria, the QoE was considered high in five outcomes, moderate in six outcomes and low in one outcome (Table S3). We assessed publication bias using a funnel plot and Egger’s test. Publication bias was detected only in percentages of ISRs. However, this meta-analysis included less than 10 studies, which limits the reliability of these publication bias assessments (Table S5).

## Discussion

This meta-analysis provides a comprehensive evaluation of the efficacy and safety of LAA compared to SOT as an HIV treatment. We found that although LAA had non-inferior efficacy to SOT, LAA was associated with a higher risk of drug resistance, which to some extent could lead to cross-resistance. This is especially important because this could limit the use of the current global first-line antiretroviral regimen that is INSTI-based. Concerning results such as these have led to questions about whether treatments should still be considered non-inferior if inadequacy is detected from another aspect, such as drug resistance.^35^ Safety analyses showed that LAA was associated with higher risks of AEs and a lower rise in CD4+ cell count than SOT.

Studies included in this meta-analysis had relatively restrictive inclusion and exclusion criteria and may not represent the wider population of PLWH. The percentage of participants excluded from screening ranged from 12% to 51%.^28–33^ The clinical trials excluded people with hepatitis B coinfection, people living with HIV-2 and pregnant women, limiting the evidence for the use of LAA in these populations.

Most trials also excluded people for whom previous ART had failed and those with a history of drug resistance. The exception was LATITUDE, which included people with a history of virological failure. Furthermore, participants in the included studies were not representative of the worldwide HIV epidemic. The majority of PLWH are women (53%) and people living in Africa (66%).^1,36^ In the included studies, only 30% of participants were women, and 41% were of Black ethnicity or African heritage. In regimens intended for global use, participants should be a representative of demographic of the global PLWH population. A representative sample should have reflected the demographic of PLWH which mostly women or girls and PLWH in LMICs. Poor representativeness in the sample could lead to biased efficacy and safety, leading to poor validity and generalizability of the results.^37,38^

LAA may be more acceptable to PLWH compared to SOT. Data in diverse populations suggest that LAA is preferred over SOT due to lower dosing frequency and reduced pill burden.^39–42^ However, LAA administration requires people to find a healthcare facility and require trained personnel to perform the injection, particularly because cabotegravir/rilpivirine is given intramuscularly in the gluteal muscle, which has particular risks due to the proximity of blood vessels and nerves.^43–45^

This meta-analysis identified that LAA is associated with increased AEs even after excluding ISRs compared to SOT, as well as greater rises in LDL. Although the reason for this phenomenon remains unclear, it may be driven by the discontinuation of TDF when switching to LAA. Evidence shows that TDF is associated with weight loss and reduced metabolic markers such as lipids and glucose.^20,46^

### Comparison with previous studies

These results were broadly consistent with another meta-analysis involving 10 957 participants from 12 included studies conducted by Wang *et al.* in 2022. However, their meta-analysis has not analysed the treatment-emergent INSTI resistance compared to SOT. In addition, 4 of their 12 included studies had either unclear or high risk of bias.^47^ Regarding safety, the results also align with the meta-analysis of Lazarus *et al.* in 2020. However, their comparator was a placebo, and their study sample size was small; there were only 666 participants from five included studies.^48^

This meta-analysis also had consistent efficacy and HIV RNA ≥ 50 copies/mL results with several observational studies, such as the OPERA cohort, a multicentre prospective observational study in Switzerland, and the BEYOND study.^49–51^ These studies reported efficacy between 92% and 99% and confirmed virological failure between 0% and 1.6%.^49–51^ However, these studies were single-arm studies without comparison and were conducted in the USA and Switzerland.^49–51^

As a weakness, in comparison to a meta-analysis conducted by Wang *et al.* in 2022, this meta-analysis only considered studies with a low risk of bias. While this approach ensures higher-quality data, it may limit the inclusiveness of the results. Incorporating all studies, irrespective of their risk of bias, could provide a more comprehensive view. On the other hand, the risk of bias could be addressed through a sensitivity analysis to show how studies with a high risk of bias impact the overall results.^47^ The other weakness was that this meta-analysis only analysed the efficacy in Week 48, while Wang *et al.* also analysed the efficacy in Week 96. While analysing the efficacy in Week 48 was considered enough, examining it in longer intervals can help understand the dynamic of LAA efficacy.^47^

### Future research

This meta-analysis highlights several avenues for future research. We identified a relatively high rate of ISR caused by cabotegravir/rilpivirine combination. As a lifetime medication, side effects like ISR could discourage PLWH from continuing LAA. Therefore, replacing rilpivirine with another candidate with potentially lower side effects and longer dosing intervals is promising, for instance, by combining cabotegravir with the novel capsid inhibitor lenacapavir.^52^ This is particularly important as no generic rilpivirine is currently available, limiting the affordability of cabotegravir/rilpivirine. We also found that current LAA studies do not reflect the broader population of PLWH. Therefore, future clinical trials should have wider inclusion and exclusion criteria that reach more diverse populations. Our findings suggest research to focus on evaluating quality of life and possible increase of life expectancy among PLWH using LAA is warranted to further inform consideration of broader implementation of LAA in the future.

### Limitations of the review

This meta-analysis had several limitations. First, this meta-analysis only considered studies with a low risk of bias or some concerns. While this approach ensures higher-quality data, it may limit the comprehensiveness of results. Second, this meta-analysis analysed efficacy at Week 48. Analyses of efficacy after longer follow-up periods could better reflect real-world scenarios.^47^ Third, we also identified high heterogeneity in measured outcomes. Despite using random-effects models to address this issue, it remains a potential source of bias and could limit the generalizability of findings.

### Conclusions

In conclusion, this meta-analysis showed that LAA has non-inferior efficacy compared to SOT. However, the implementation of LAA should carefully consider the potential higher risk of developing drug resistance and INSTI cross-resistance than those who received SOT. LAA was also associated with an increased risk of AEs, including those leading to treatment discontinuation.

While the role of LAA in HIV treatment is promising, there remain several significant challenges, not least access, and affordability. More research is urgently needed to understand the role of LAA in a broader population, including people with hepatitis B coinfection, people living with HIV-2 and pregnant women.

## Supplementary Material

dkae480_Supplementary_Data

## Supplementary data

Figures S1 to S11, Tables S1 to S5 and Appendices S1 and S2 are available as Supplementary data at *JAC* Online.
