# Supplementary material for: Safety and efficacy of long-acting cabotegravir/rilpivirine versus standard oral antiretroviral therapy: a systematic review and meta-analysis
Source: J Antimicrob Chemother. 2025 Jan 8;80(3):624–32. doi: 10.1093/jac/dkae480 (PMC11879206; doi:10.1093/jac/dkae480)
Supplement: dkae480_Supplementary_Data [file dkae480_supplementary_data.docx]

# **SUPPLEMENTARY MATERIAL**

## **Appendix S1. Strategy used for systematic literature search in Embase and PubMed**

| **Search terms used in Embase** | | | |
| --- | --- | --- | --- |
| **#** | **Query** | | **Keyword** |
| 1 | ('human immunodeficiency virus' or (human and immunodeficiency and virus) or (acquired and immune and deficiency and syndrome) or 'acquired immune deficiency syndrome virus' or aids or hiv or 'hiv 1' or 'hiv 2' or hiv1 or hiv2 or 'human immune deficiency virus' or 'human immuno-deficiency virus' or 'human immune-deficiency virus' or 'acquired immuno-deficiency syndrome' or 'acquired immune-deficiency syndrome' or 'hivaids' or 'hiv positive' or hiv or 'hiv aids' or plwh or PWH).mp. | | HIV |
| 2 | exp Human immunodeficiency virus/ | |  |
| 3 | exp acquired immune deficiency syndrome/ | |  |
| 4 | exp HIV survivor/ | |  |
| 5 | exp Human immunodeficiency virus infection/ | |  |
| 6 | exp Human immunodeficiency virus 1 infection/ | |  |
| 7 | human immunodeficiency virus 1.mp. | |  |
| 8 | exp Human immunodeficiency virus prevalence/ | |  |
| 9 | 1 or 2 or 3 or 4 or 5 or 6 or 7 or 8 | |  |
| 10 | exp drug efficacy/ | | Efficacy |
| 11 | drug effectiveness.mp. | |  |
| 12 | drug effectivity.mp. | |  |
| 13 | pharmacologic* effectiveness.mp. | |  |
| 14 | pharmacologic* efficacy.mp. | |  |
| 15 | clinical effect.mp. | |  |
| 16 | clinical benefit.mp. | |  |
| 17 | exp clinical outcome/ | |  |
| 18 | therapeutic efficacy.mp. | |  |
| 19 | exp therapy effect/ | |  |
| 20 | therapeutic effect.mp. | |  |
| 21 | therapeutic benefit.mp. | |  |
| 22 | therapeutic outcome.mp. | |  |
| 23 | treatment effectiveness.mp. | |  |
| 24 | exp treatment outcome/ | |  |
| 25 | 10 or 11 or 12 or 13 or 14 or 15 or 16 or 17 or 18 or 19 or 20 or 21 or 22 or 23 or 24 | |  |
| 26 | exp drug safety/ | | Safety |
| 27 | exp safety/ | |  |
| 28 | exp adverse drug reaction/ | |  |
| 29 | exp drug toxicity/ | |  |
| 30 | exp adverse event/ | |  |
| 31 | exp drug tolerability/ or exp side effect/ | |  |
| 32 | treatment complication.mp. | |  |
| 33 | safety profile.mp. | |  |
| 34 | adverse drug effect.mp. | |  |
| 35 | adverse drug event.mp. | |  |
| 36 | drug adverse effect.mp. | |  |
| 37 | drug adverse reaction.mp. | |  |
| 38 | drug side effect.mp. | |  |
| 39 | drug-related adverse event.mp. | |  |
| 40 | drug-related adverse effect.mp. | |  |
| 41 | drug-related adverse reaction.mp. | |  |
| 42 | 26 or 27 or 28 or 29 or 30 or 31 or 32 or 33 or 34 or 35 or 36 or 37 or 38 or 39 or 40 or 41 | |  |
| 43 | exp drug resistance/ | | Resistance |
| 44 | exp antiviral resistance/ | |  |
| 45 | exp treatment failure/ | |  |
| 46 | exp breakthrough infection/ | |  |
| 47 | drug resistance.mp. | |  |
| 48 | antiviral resistance.mp. | |  |
| 49 | treatment failure.mp. | |  |
| 50 | breakthrough infection.mp. | |  |
| 51 | virologic* failure.mp. | |  |
| 52 | viral failure.mp. | |  |
| 53 | incomplete virological response.mp. | |  |
| 54 | incomplete viral response.mp. | |  |
| 55 | mutation*.mp. | |  |
| 56 | exp mutation/ | |  |
| 57 | drug resistan* mutation*.mp. | |  |
| 58 | resistance.mp. | |  |
| 59 | hivdr.mp. | |  |
| 60 | antiretroviral resistan*.mp. | |  |
| 61 | 43 or 44 or 45 or 46 or 47 or 48 or 49 or 50 or 51 or 52 or 53 or 54 or 55 or 56 or 57 or 58 or 59 or 60 | |  |
| 62 | exp Long acting drug/ | | Long-acting antiretroviral |
| 63 | long-acting antiretroviral.mp. | |  |
| 64 | long acting agent*.mp. | |  |
| 65 | long-acting injectable*.mp. | |  |
| 66 | long-acting agent*.mp. | |  |
| 67 | long-acting ART.mp. | |  |
| 68 | extended-release antiretroviral.mp. | |  |
| 69 | exp sustained drug release/ | |  |
| 70 | sustained-release antiretroviral.mp. | |  |
| 71 | (long and acting and injectable*).mp. | |  |
| 72 | exp cabotegravir/ | |  |
| 73 | exp cabotegravir plus rilpivirine/ | |  |
| 74 | cabotegravir.mp. | |  |
| 75 | apretude.mp. | |  |
| 76 | cabotegravir sodium.mp. | |  |
| 77 | gsk 1265744.mp. | |  |
| 78 | gsk 1265744a.mp. | |  |
| 79 | gsk 744.mp. | |  |
| 80 | gsk1265744.mp. | |  |
| 81 | vocabria.mp. | |  |
| 82 | CAB.mp. | |  |
| 83 | CAB-LA.mp. | |  |
| 84 | CABENUVA.mp. | |  |
| 85 | exp lenacapavir/ | |  |
| 86 | lenacapavir sodium.mp. | |  |
| 87 | Sunlenca.mp. | |  |
| 88 | gs 6207.mp. | |  |
| 89 | gs ca 1.mp. | |  |
| 90 | gs ca1.mp. | |  |
| 91 | gs ca2.mp. | |  |
| 92 | gs-hiv.mp. | |  |
| 93 | gs6207.mp. | |  |
| 94 | exp islatravir/ | |  |
| 95 | mk 8591.mp. | |  |
| 96 | mk8591.mp. | |  |
| 97 | MK-8591.mp. | |  |
| 98 | 62 or 63 or 64 or 65 or 66 or 67 or 68 or 69 or 70 or 71 or 72 or 73 or 74 or 75 or 76 or 77 or 78 or 79 or 80 or 81 or 82 or 83 or 84 or 85 or 86 or 87 or 88 or 89 or 90 or 91 or 92 or 93 or 94 or 95 or 96 or 97 | |  |
| 99 | 9 and 25 and 42 and 61 and 98 | |  |
| 100 | 9 and 25 and 42 and 98 | |  |
| 101 | 9 and 61 and 98 | |  |
| 102 | 99 or 100 or 101 | | Complete search |
|  | | | |
| **Search terms used in PubMed** | | | |
| **#** | | **Query** | **Keyword** |
| 1 | | “human immunodeficiency virus” OR (human AND immunodeficiency AND virus) OR (acquired AND immune AND deficiency AND syndrome) OR ”acquired immune deficiency syndrome virus” OR aids OR hiv OR ”hiv 1” OR ”hiv 2” OR hiv1 OR hiv2 OR ”human immune deficiency virus” OR ”human immuno-deficiency virus” OR ”human immune-deficiency virus” OR ”acquired immuno-deficiency syndrome” OR ”acquired immune-deficiency syndrome” OR ”hivaids” OR ”hiv positive” OR hiv OR ”hiv aids” OR plwh OR “HIV”[MeSH] OR "HIV Long-Term Survivors"[MeSH] OR "HIV Infections"[MeSH] OR "HIV-2"[MeSH] OR "HIV-1"[MeSH] OR "HIV Seropositivity"[MeSH] OR "acquired immunodeficiency syndrome"[MeSH] | HIV |
| 2 | | “long acting drug” OR “long-acting antiretroviral” OR “long acting agent*” OR “long-acting injectable*” OR “long-acting agent*” OR “long-acting ART” OR “extended-release antiretroviral” OR “sustained drug release” OR (long AND acting AND injectable*) OR “cabotegravir” OR “apretude” OR “cabotegravir sodium” OR “gsk 1265744” OR “gsk 744” OR “gsk1265744” OR “gsk1265744a” OR “vocabria” OR “CAB” OR “CAB-LA” OR “lenacapavir” OR “Sunlenca” OR “gs 6207” OR “gs ca1” OR “gs ca2” OR “gs-hiv” OR “gs6207” OR “Islatravir” OR “mk 8591” OR “mk8591” OR “MK-8591” OR “MK8591” | Long-acting antiretroviral |
| 3 | | “treatment outcome” [MeSH] OR “outcome, treatment” OR “treatment efficacy” OR “efficacy, treatment” OR “clinical efficacy” OR “efficacy, clinical” OR “clinical effectiveness” OR “effectiveness, clinical” OR “treatment effectiveness” OR “effectiveness, treatment” OR “treatment outcome” | Efficacy |
| 4 | | “drug-related side effects and adverse reactions”[MeSH] “adverse drug event” OR “adverse drug events” OR “adverse drug reaction” OR “adverse drug reactions” OR “drug event, adverse” OR “drug events, adverse” OR “drug reaction, adverse” OR “drug reactions, adverse” OR “drug related side effects and adverse reaction” OR “drug related side effects and adverse reactions” OR “drug side effect” OR “drug side effects” OR “drug toxicities” OR “drug toxicity” OR “reactions, adverse drug” OR “side effect, drug” OR “side effects, drug” OR “side effects of drugs” “toxicities, drug” OR “toxicity, drug” OR “safety” | Safety |
| 5 | | “drug resistance, viral” [MeSH] OR “treatment failure”[MeSH] OR “drug resistance” OR “antiviral resistance” OR “treatment failure” OR “breakthrough infection” OR “virological failure” OR “viral failure” OR “incomplete virological response” OR “incomplete viral response” OR “mutation*” OR “drug resistan* mutation*” OR resistance | Resistance |
| 6 | | #1 AND #2 AND #3 AND #4 AND #5 |  |
| 7 | | #1 AND #2 AND #3 AND #4 |  |
| 8 | | #1 AND #2 AND #5 |  |
| 9 | | #6 OR #7 OR #8 | Complete search |

##

## **Appendix S2. Data extraction form**

| **No.** | **Trial name** | **Treatment or prevention** | **Study identifiers** | | | | | | | | |
| --- | --- | --- | --- | --- | --- | --- | --- | --- | --- | --- | --- |
|  |  |  | **Year** | **Author** | **Link to study** | **Source** | **Trial ID** | **Type** | **ITT/PP** | **Countries involved** | **Length of F/u** |
|  |  |  |  |  |  |  |  |  |  |  |  |
|  |  |  |  |  |  |  |  |  |  |  |  |

| **Population characteristics (Study Demographics)** | | | | | | | |
| --- | --- | --- | --- | --- | --- | --- | --- |
| **Eligibility criteria** | **Age (mean/median, and range/SD)** | **Female sex, No (ITT%)** | **Ethnicity breakdown, No/total (ITT%)** | **Race breakdown, No/total (ITT%)** | **Baseline CD4+ count** | **ART history** | **Resistance status** |
|  |  |  |  |  |  |  |  |
|  |  |  |  |  |  |  |  |

| **Intervention** | | | | **Comparator** | | | | **Treatment Efficacy, No/total (ITT%)** | | | |
| --- | --- | --- | --- | --- | --- | --- | --- | --- | --- | --- | --- |
| **Regimen** | **Dose** | **Route** | **Total participants** | **Regimen** | **Dose** | **Route** | **Total participants** | **HIV RNA <50 copies/ml** | | **HIV RNA ≥50 copies/ml** | |
|  |  |  |  |  |  |  |  | **LAA** | **SOT** | **LAA** | **SOT** |
|  |  |  |  |  |  |  |  |  |  |  |  |
|  |  |  |  |  |  |  |  |  |  |  |  |

| **Prevention Efficacy** | | | | **Safety, No/total (ITT%)** | | | | | | | | | | | |
| --- | --- | --- | --- | --- | --- | --- | --- | --- | --- | --- | --- | --- | --- | --- | --- |
| **New HIV infection, No/total (ITT%)** | | **Incidence rate, (95% CI)** | | **Gr 1-4 AE** | | **Gr 1-4 AE excluding ISR** | | **ISR** | | **Discontinuation of treatment for AE** | | **LDL increased** | | **Glucose increased** | |
| **LAA** | **SOT** | **LAA** | **SOT** | **LAA** | **SOT** | **LAA** | **SOT** | **LAA** | **SOT** | **LAA** | **SOT** | **LAA** | **SOT** | **LAA** | **SOT** |
|  |  |  |  |  |  |  |  |  |  |  |  |  |  |  |  |
|  |  |  |  |  |  |  |  |  |  |  |  |  |  |  |  |

| **Safety** | | | | **Resistance, No/total (ITT%)** | | | | | |
| --- | --- | --- | --- | --- | --- | --- | --- | --- | --- |
| **Weight change from baseline** | | **CD4+ change from baseline** | | **Protocol defined VF** | | **Successful genotyped** | | **INSTI-associated DRMs** | |
| **LAA** | **SOT** | **LAA** | **SOT** | **LAA** | **SOT** | **LAA** | **SOT** | **LAA** | **SOT** |
|  |  |  |  |  |  |  |  |  |  |
|  |  |  |  |  |  |  |  |  |  |

Abbreviations: ITT, Intention-to-treat; PP, Per-protocol; SD, Standard deviation; ART, Antiretroviral therapy; LAA, Long-acting antiretroviral; SOT, Standard oral treatment; Gr, Grade; AE, Adverse events; ISR, Injection site reaction; LDL, Low-density lipoprotein; VF, Virologic failure

| **Table S1. Summary table of participants’ characteristics from all included trials** | | | |
| --- | --- | --- | --- |
| **Study** | **Median age (range)** | **Black or African heritage, No/total (%)** | **Female, No/total (%)** |
| FLAIR | 34 (18-68) | 103/566 (18) | 127/566 (22) |
| LATTE-2 | 35 (19-64) | 32/171 (18.7) | 18/171 (10.5) |
| SOLAR | 37 (18-74) | 144/670 (21) | 118/670 (18) |
| ATLAS | 42 (18-82) | 139/616 (23) | 203/616 (33) |
| CARES | 43 (35-51) | 510/512 (99.6) | 295/512 (57.6) |
| LATITUDE* | 40 (32,51) ^#^ | 277/434 (64) | 129/434 (30) |
| **Total** |  | 1205/2969 (40.6) | 890/2969 (30) |
| *****Data based on step 1 or before randomization of eligible participants  ^#^Interquartile range (IQR) | | | |

| **Table S2. Risk of bias assessment using RoB tolos II by Cochrane** | | | | | | | |
| --- | --- | --- | --- | --- | --- | --- | --- |
| **Study name** | **D1** | **D2** | **D3** | **D4** | **D5** | **Overall** |  |
| FLAIR |  |  |  |  |  |  | Low risk |
| SOLAR |  |  |  |  |  |  | Some concerns |
| ATLAS |  |  |  |  |  |  | High risk |
| CARES |  |  |  |  |  |  |  |
| LATTE-2 |  |  |  |  |  |  |  |
| LATITUDE |  |  |  |  |  |  |  |

**Table S3. Summary table of evidence based on GRADE**

| **Population:** PLWH of any age, background, or geographic region  **Intervention:** Long-acting antiretroviral two-drug regimen (LAA-2DR)  **Comparator:** Standard oral treatment (SOT) | | | | | |
| --- | --- | --- | --- | --- | --- |
| **Outcome** | **Trial arm** | | **Effect** | | **GRADE certainty of evidence** |
|  | **LAA-2DR**  **n (%)** | **SOT**  **n (%)** | **Relative**  **(95% CI)** | **Absolute**  **(95% CI)** |  |
| HIV RNA <50 copies/ml at week 48 | 1422/1554 (91.5%) | 1166/1275 (91.5%) | No difference in suppressing HIV RNA <50 copies/ml (risk difference, -0.00; -0.03 to 0.02) | | **High** |
| Treatment-emergent INSTI resistance in successfully-genotypes participants | 11/18 (61.1%) | 0/29 (0.0%) | INSTI resistance in LAA-2DR (meta-analysis pooled estimate, 57% (33%-78%) was higher compared to SOT (meta-analysis pooled estimate, 9% (2%-30%) | | **Moderate** |
| Grade 1-4 AEs | 1301/1408 (92.4%) | 830/1127 (73.6%) | RR 1.22  (1.12 to 1.33) | 162 (88-243) more AEs per 1000 PLHIV with LAA-2DR than with SOT | **Moderate** |
| AEs leading to treatment discontinuation | 50/1408 (3.6%) | 15/1127 (1.3%) | RR 2.58  (1.43 to 4.67) | 21 (6-49) additional AEs leading to treatment discontinuation per 1,000 PLHIV in LAA-2DR compared to SOT | **High** |
| Incident hypertension | 41/961 (4.3%) | 23/904 (2.5%) | RR 1.75 (1.07 to 2.87) | 19 (2-48) additional cases of incident hypertension with LAA-2DR compared to SOT | **High** |
| Weight change from baseline | 842 | 842 | - | MD 0.94 (-0.14-2.02) kg greater weight gain in LAA-2DR compared to SOT | **Moderate** |
| CD4+ level change from baseline | 1256 | 1071 | - | MD rise in CD4 count was 21.6 (37.97- 5.24) cells/mm3 lower in LAA-2DR compared to SOT | **High** |
| LDL level change from baseline | 552 | 568 | - | MD rise in LDL level was 0.22 (0.06-0.38) mmol/L higher with LAA-2DR compared to SOT | **Moderate** |
| Glucose level change from baseline | 837 | 808 | - | MD rise in glucose level was 0.03 (0.06-0.13) mmol/L higher with LAA-2DR compared to SOT | **High** |
| HIV RNA ≥50 copies/ml | 2.3% (36/1554) | 3.5% (45/1,275) | No difference in RNA ≥50 copies/ml (risk difference -0.00; -0.03 to 0.02) | | **Moderate** |
| Grade 1-4 AEs excluding ISR | 1039/1293 (80.4%) | 778/1071 (72.6%) | RR 1.10 (1.03 to 1.19) | 73 (22-138) additional Gr1-4 AEs excluding ISR per 1,000 with LAA-2DR compared to SOT | **Moderate** |
| Injection site reactions | 79% (1,108/1,408) | | Injection site reaction incidence (percentage pooled estimate) was 83% (71%-90%) | | **Low** |
| Abbreviations: CI, confidence interval; RR, risk ratio; MD, mean difference; INSTI, integrase strand transfer inhibitor | | | | | |


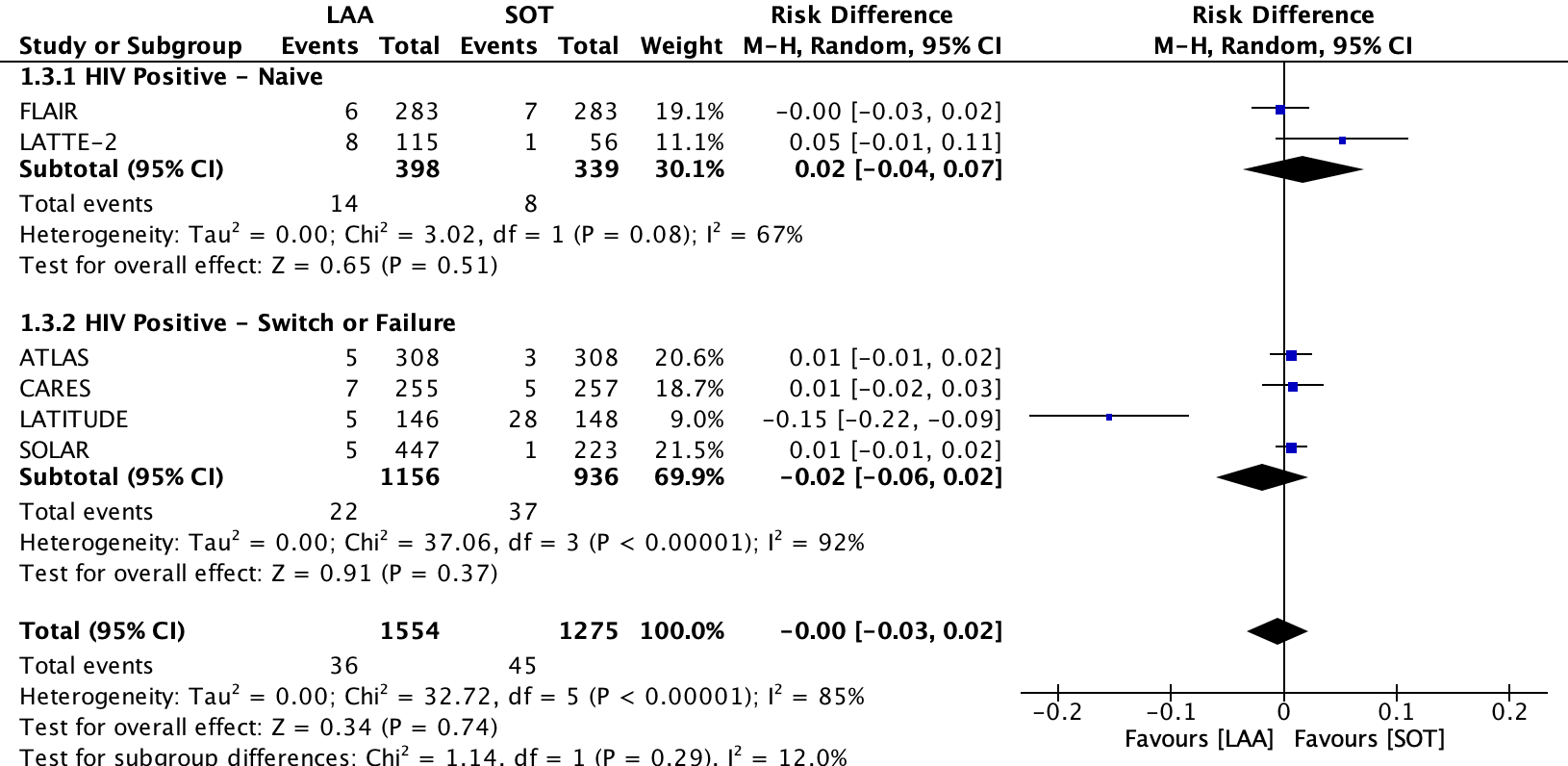


Figure S1. Forest plot for HIV RNA ≥50 copies/ml at week 48, LAA vs SOT


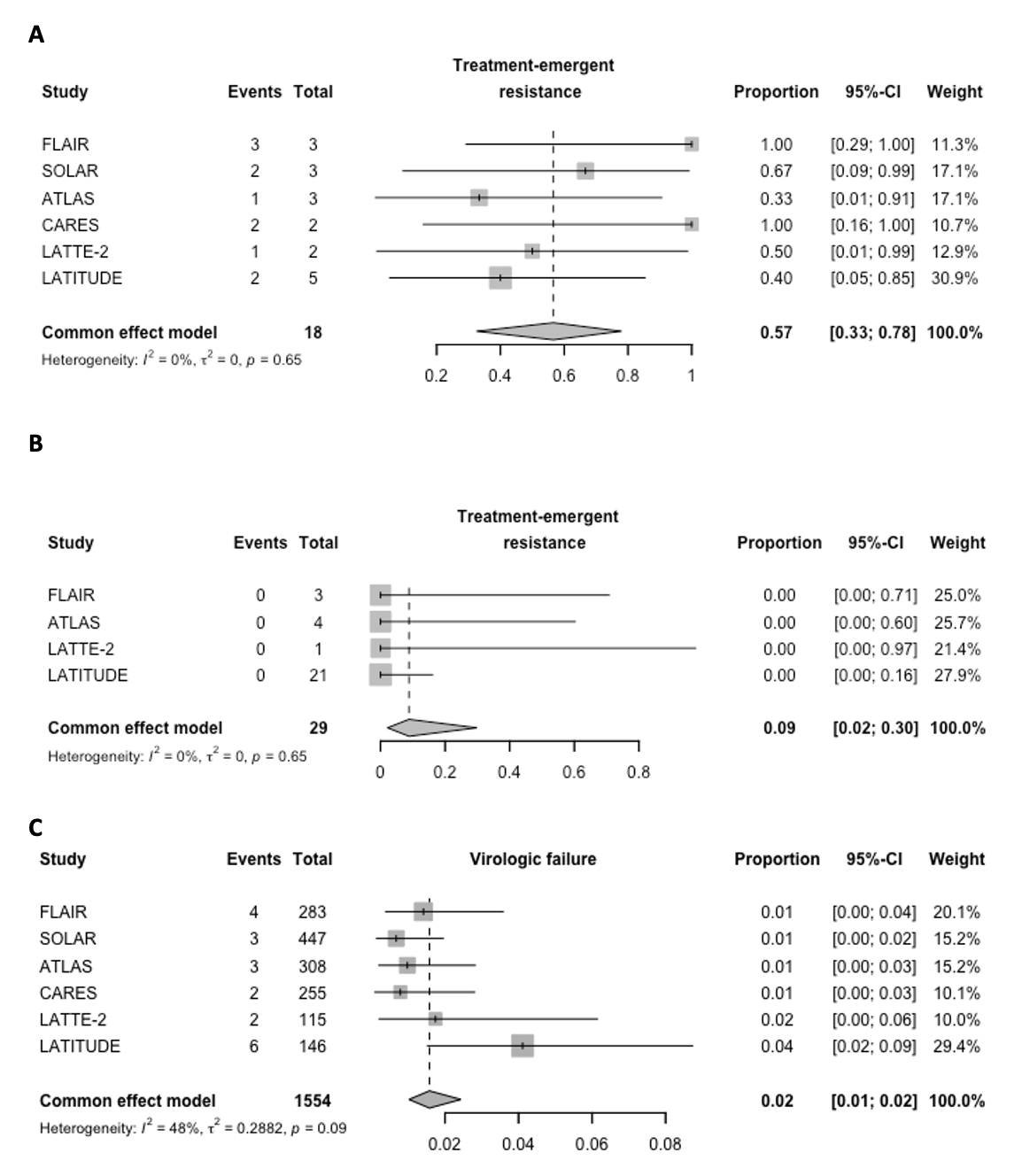


Figure S2. Forest plot for treatment-emergent INSTI resistance in LAA (2A) and SOT (2B) among successfully genotyped participants. The proportion of 0.09 resulted from statistical calculations that applied continuity correction by adding 0.5 to both numerator and denominator rather than the raw value of 0.

| **Table S4. INSTI resistance level and cross-resistance** | | | | |
| --- | --- | --- | --- | --- |
| **Study** | **Participant** | **INSTI-associated DRMs** | **CAB resistance** | **DTG cross resistance** |
| **Treatment** | | | | |
| FLAIR | 1 | G140R | High | Potential low |
|  | 2 | Q148R | High | Low |
|  | 3 | Q148R | High | Low |
| SOLAR | 1 | Q148R | High | Low |
|  | 2 | G118R | High | High |
| ATLAS | 1 | N155H | Intermediate | Potential low |
| CARES | 1 | E92E/V; N155H; L74M* | Intermediate | Potential low |
|  | 2 | G118R | High | High |
| LATTE-2 | 1 | Q148R | High | Low |
| LATITUDE | 1 | E138EK; G140GS; Q148K | High | High |
|  | 2 | E138K; Q148K | High | Intermediate |
| **Prevention** | | | | |
| HPTN 083 | 1 | R263K | High | Intermediate |
|  | 2 | Q148R | High | Low |
| *INSTI accessory mutation; CAB: cabotegravir; DTG: dolutegravir  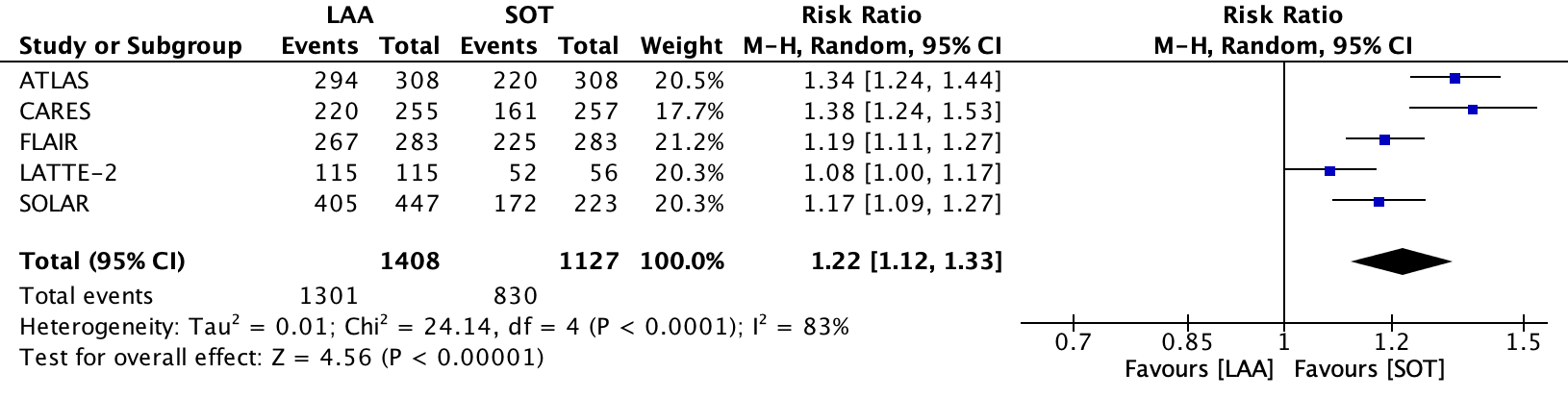  Figure S3. Forest plot for grade 1-4 AEs, LAA vs SOT  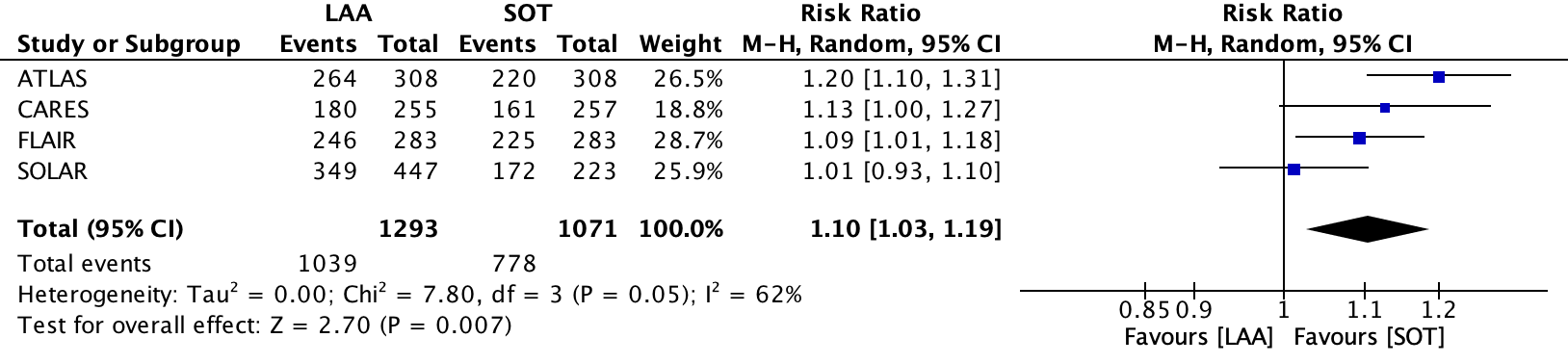  Figure S4. Forest plot for grade 1-4 AEs excluding ISR, LAA vs SOT  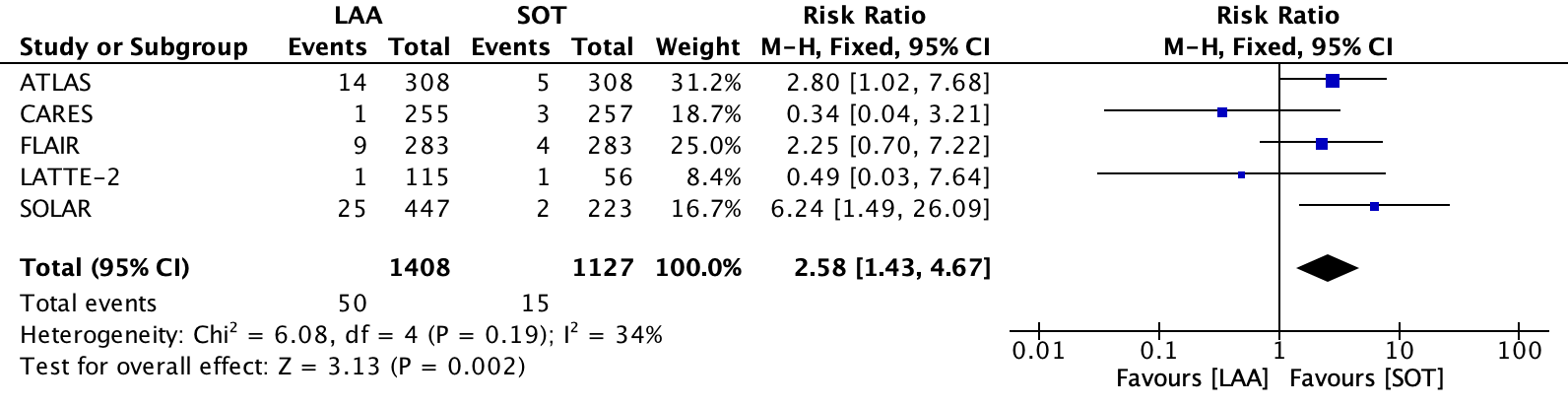  Figure S5. Forest plot for discontinuation of treatment for AEs  **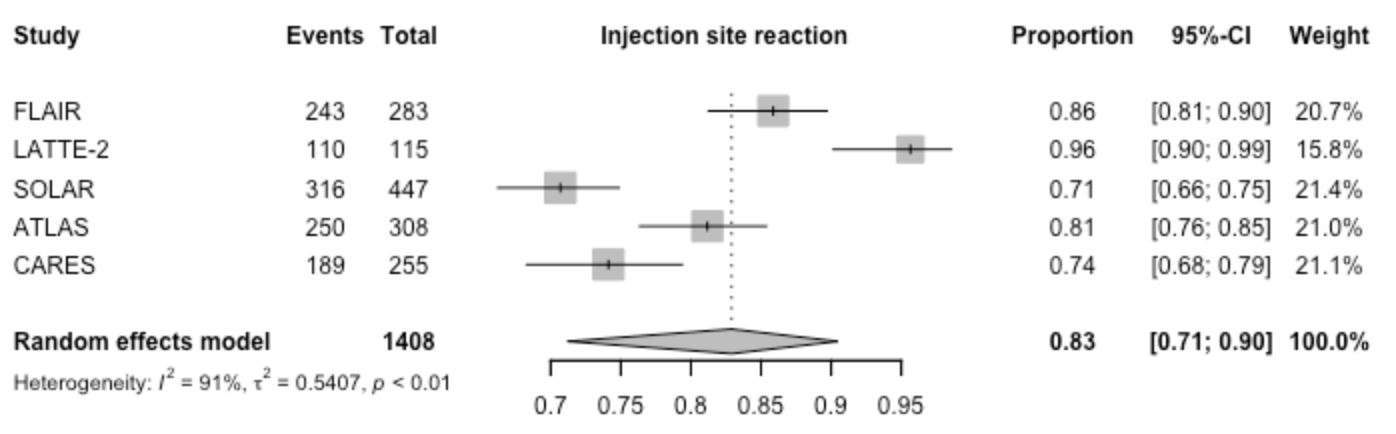**  Figure S6. Forest plot for the percentage of ISR among LAA participants  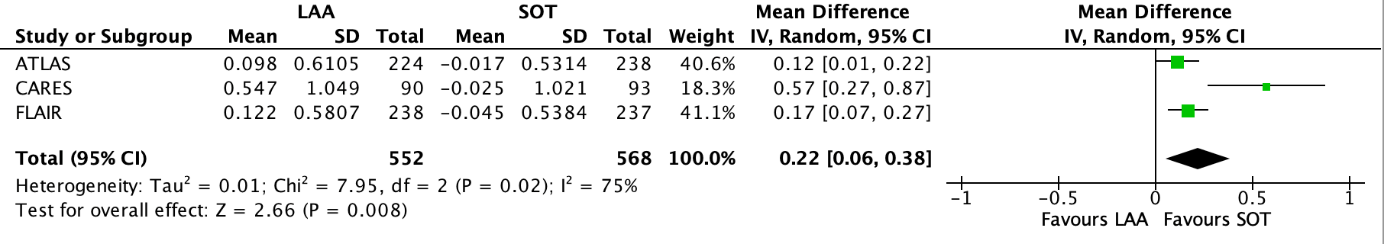  **Figure S7.** Change of LDL level from baseline  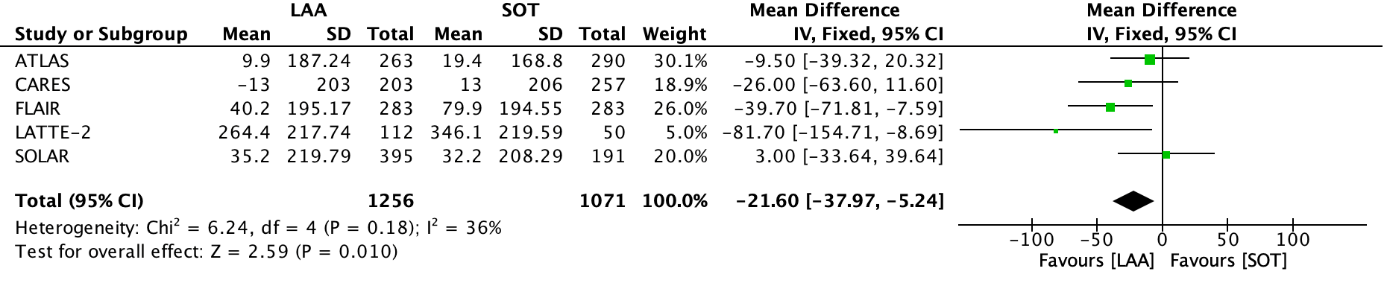  **Figure S8.** Change of CD4+ from baseline  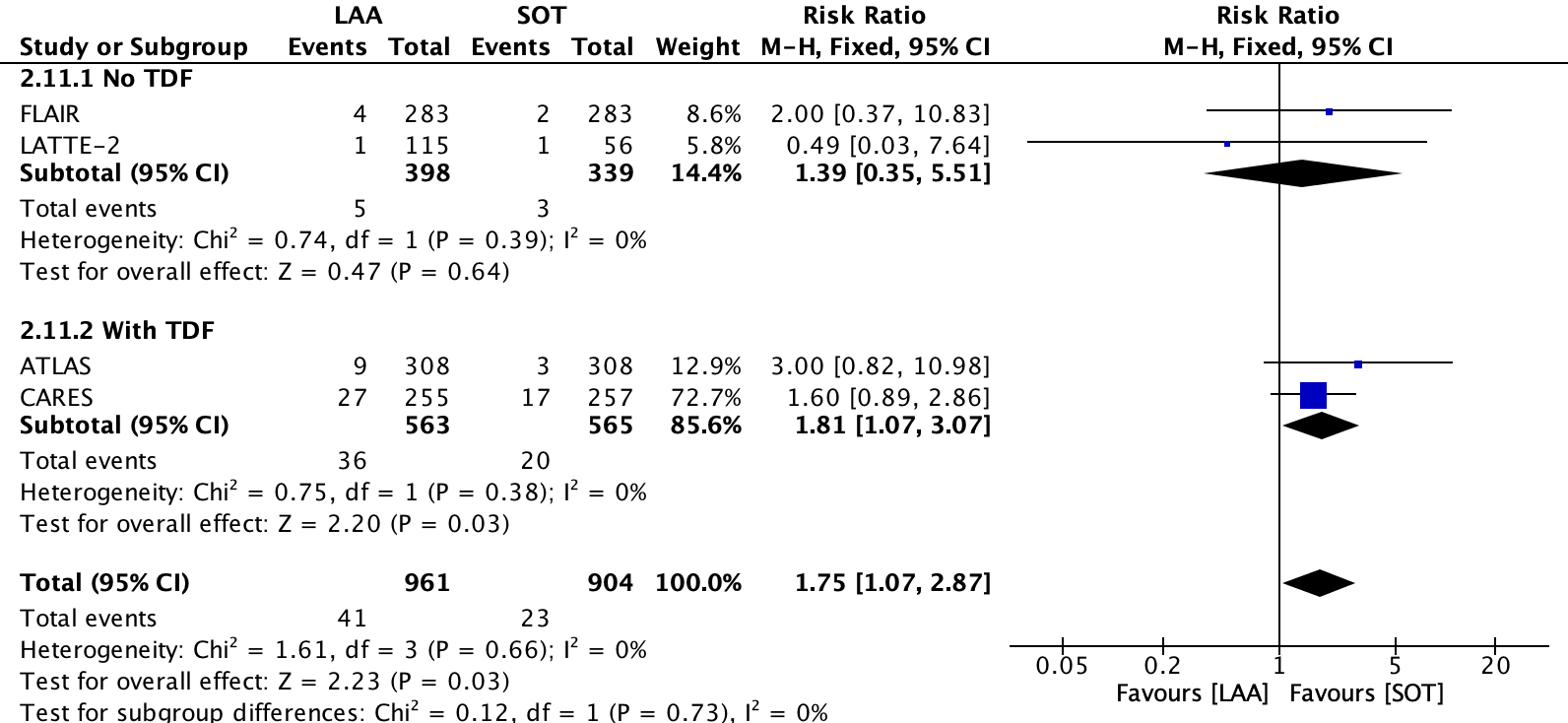  Figure S9. Forest plot for hypertension, LAA vs SOT | | | | |


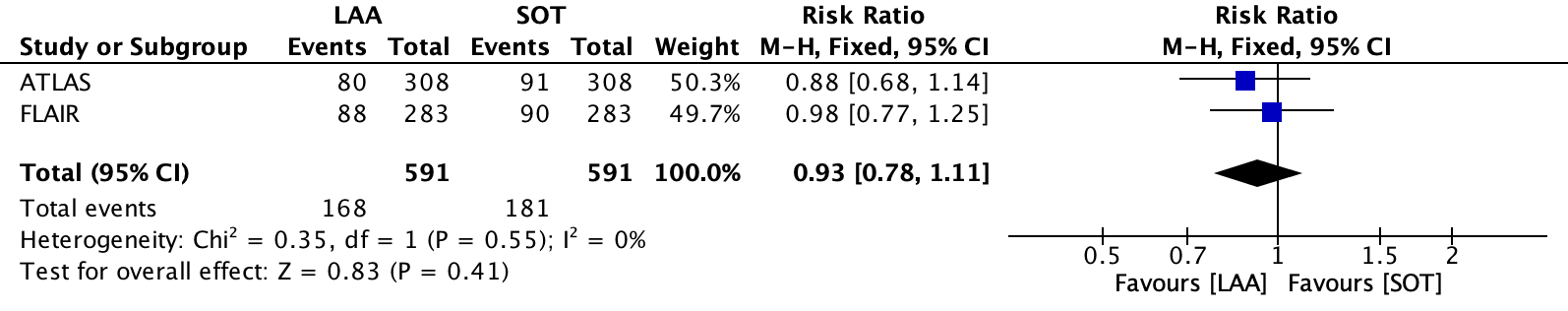


Figure S10. Forest plot showing change in glucose, LAA vs SOT


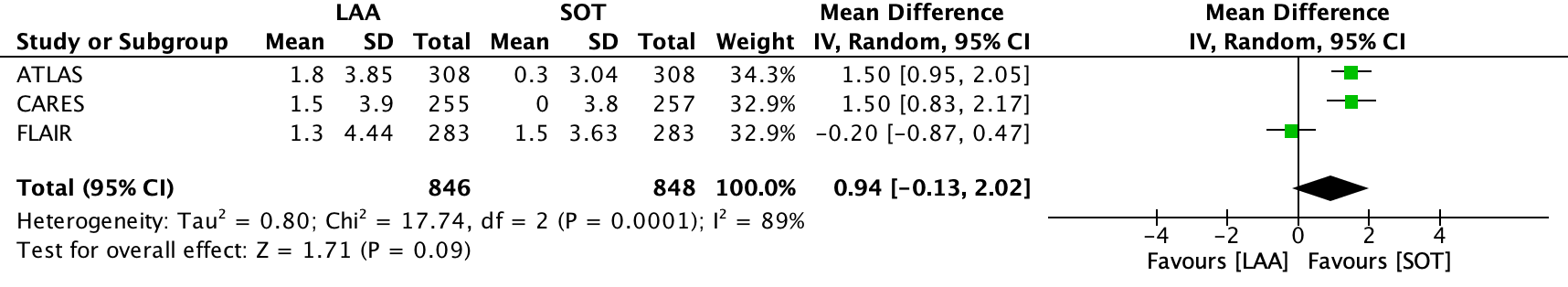


Figure S11. Forest plot showing change in Weight, LAA vs SOT

**Table S5. Assessment of publication bias**

| **Outcome** | **Funnel plot** | **Egger’s test** | **Publication bias** |
| --- | --- | --- | --- |
| HIV RNA <50 copies/ml at week 48 | 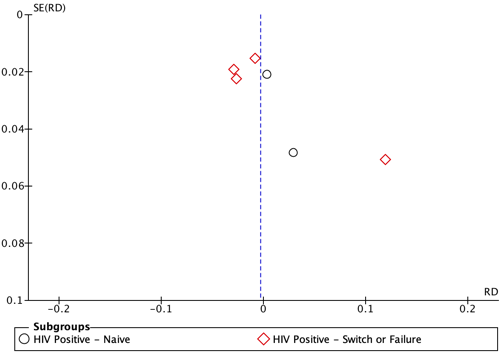 | *p =* 0.1065 | Not detected |
| Treatment-emergent INSTI resistance | 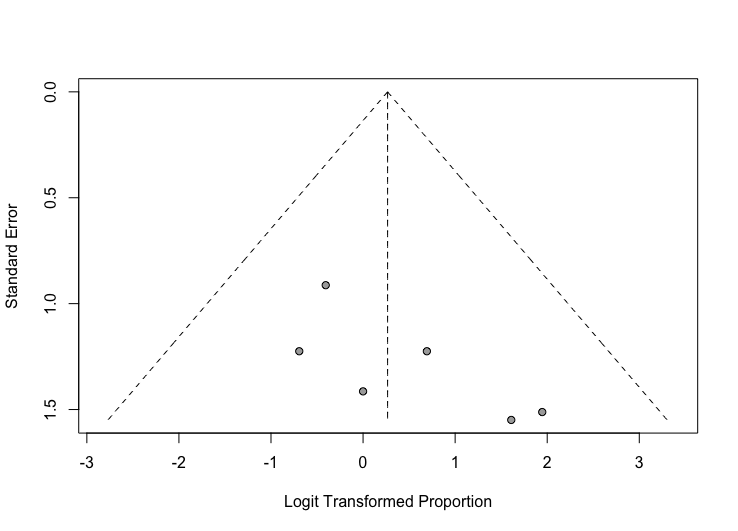 | *p =* 0.0956 | Not detected |
| Grade 1-4 adverse events | 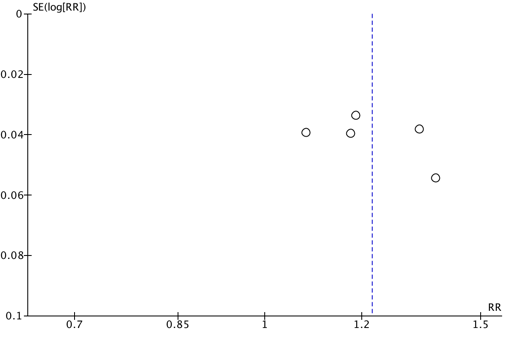 | *p =* 0.3691 | Not detected |
| Discontinuation of treatment for adverse events | 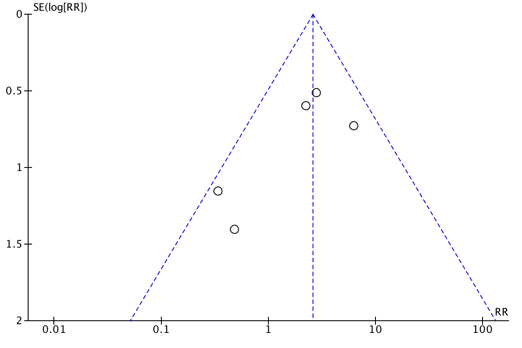 | *p =* 0.4409 | Not detected |
| Hypertension | 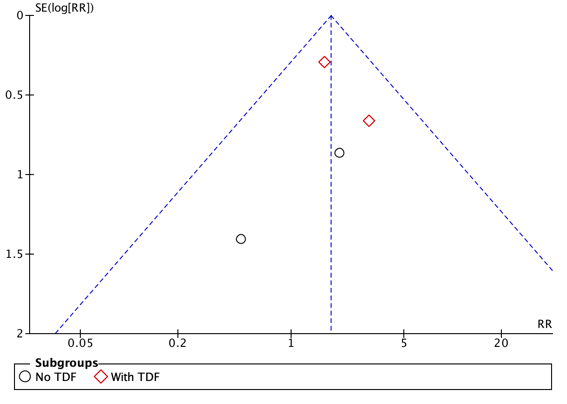 | *p =* 0.7616 | Not detected |
| Weight change from baseline | Not available | *p =* 0.6502 | Not detected |
| CD4+ level change from baseline | 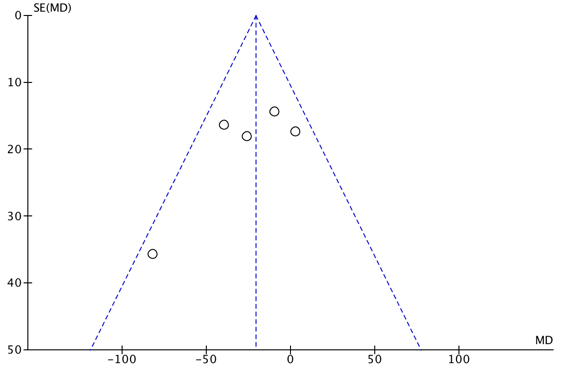 | *p =* 0.2894 | Not detected |
| Change of LDL level from baseline | Not available | *p* = 0.1871 | Not detected |
| Change of glucose level from baseline | Not available | *p* = 0.4561 | Not detected |
| HIV RNA ≥50 copies/ml | 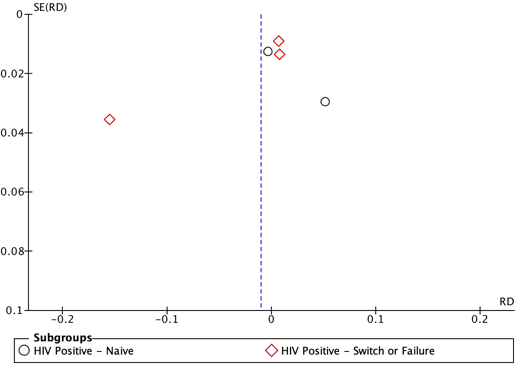 | *p* = 0.3853 | Not detected |
| Grade 1-4 AEs excluding ISR | 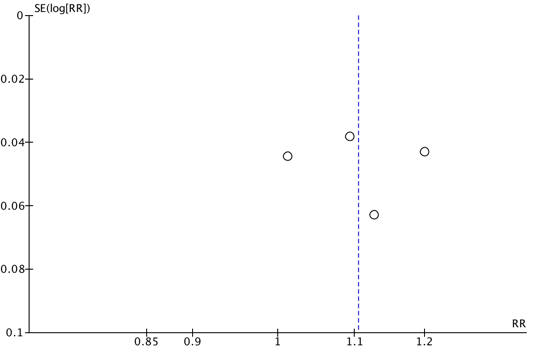 | *p* = 0.8514 | Not detected |
| Injection site reactions | 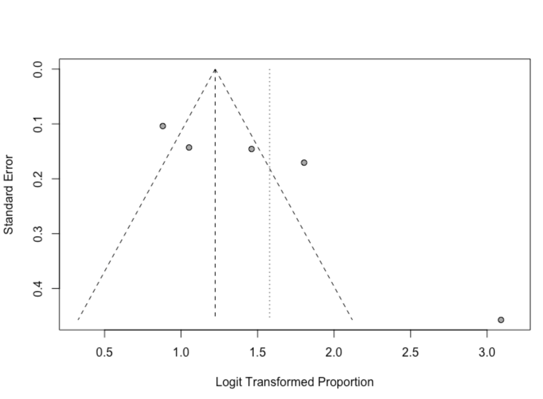 | *p =* 0.0472 | Detected |
